# New Functionalized Ionic Liquids Based on POSS for the Detection of Fe^3+^ Ion

**DOI:** 10.3390/polym13020196

**Published:** 2021-01-07

**Authors:** Wensi Li, Shengyu Feng

**Affiliations:** 1 Key Laboratory of Special Functional Aggregated Materials Ministry of Education, School of Chemistry and Chemical Engineering, Shandong University, Jinan 250100, China; anterlina@163.com; 2 National Engineering Technology Research Center for Colloidal Materials, Shandong University, Jinan 250100, China

**Keywords:** ionic liquids, thiol-ene “click” reaction, polyhedral oligomeric silsesquioxanes, fluorescent sensors

## Abstract

This study reports a novel series of imidazolium ionic liquids (ILs) based on polyhedral oligomeric silsesquioxanes (POSS) towards effective detection of metal ions, especially Fe3+ ion. ^1^H, ^13^C, ^29^Si NMR, high resolution mass spectra (HRMS) and Fourier transform infrared spectra (FTIR) were applied to confirm the structures of the ILs based on POSS (ILs-POSS). The three ILs-POSS were synthesized via a green chemistry approach—a thiol-ene “click” reaction without metal ions as catalysts. Furthermore, the spherical vesicle structures of the ILs-POSS were observed and caused by self-assembly behaviors. Through comprehensive characterizations, these ILs-POSS have performed excellent thermal stabilities and low glass transition temperatures. In addition, we found it very meaningful that the limits of detection (LODs) of the three ILs-POSS for the detection of the Fe^3+^ ion were 7.91 × 10^−8^ M, 1.2 × 10^−7^ M, and 1.2 × 10^−7^ M, respectively. These data illustrate that these ILs-POSS have great potential for the detection of the Fe^3+^ ion. In conclusion, this study not only prepared novel ILs-POSS, but also provided new materials as fluorescent sensors in the detection of Fe^3+^.

## 1. Introduction

In recent years, ionic liquids (ILs) have been widely applied in numerous chemical engineering fields such as eletrochemical sensors [1], catalysis solvents [2], capacitors [3], lithium batteries [4], and fuel cells [5] due to their distinct properties, which include high ionic conductivity, non-flammability [6], a wide electrochemical stability window [7], negligible vapor pressure, and high thermal stability [8]. ILs have attracted lots of attention owing to their regulable structures [7] composed of various organic cations and inorganic cations [9], and chosen as ideal alternatives to traditional organic solvents in the field of materials chemistry [4] and the industrial field [10]. For example, due to their high ionic conductivity [11] and wide electrochemical stability windows, ILs have been applied as actuators in the emerging fields of lithium batteries [12], fuel cells, and electrolytes [13].

However, some certain conditions have limited the development and employment of ILs. Thus, it is urgent to produce a more multifunctional and composite new class of ILs.

Over the past decades, polyhedral oligomeric silsesquioxanes (POSS) [14] have played an important role in the materials chemistry field due to their excellent properties, such as chemical stability [13] and thermal properties [15], which profit from the Si-O-Si frameworks provided with high bond energies [16]. In addition, POSS are widely compatible with varieties of organic functional groups attributed [17] to the multiple possibilities and selectivity of various kinds of organic substituent groups [15]. Furthermore, several reports have represented that POSS [16] can be employed as cores in the construction of star-shaped materials [18] and ILs containing nanobuilding blocks ascribed to the organic frameworks including Si-O-Si bonds that make POSS an ideal core [19]. Given the credit to the above advantages, POSS are supposed to be distinguished precursors in sorts of composite materials [20].

The study of the preparations and applications of ILs-POSS have been reported by many researchers over the decades. For example, Chen [21] reported a new family of imidazolium-based ionic porous hybrid polymers (termed IM-iPHPs) with polyhedral oligomeric silsesquioxane (POSS)-derived silanols (Si–OH) towards the efficient catalytic conversion of carbon dioxide (CO_2_) under mild conditions. Akbari [16] prepared two novel heterogeneous hybrid catalysts without loss of catalytic activity for at least three and six runs successfully by means of using octa-aminopropyl polyhedral oligomeric silsesquioxane hydrochloride salt (OA-POSS) and ionic liquids, followed by immobilization of Cu and Ag nanoparticles. However, the fluorescent properties of ILs-POSS in the detection of metal ions have not been discussed and the applications of ILs-POSS have not been expanded. ILs-POSS have multifunctional organic groups and low glass-transition temperatures, which profit from the structure of POSS, therefore we can take advantage of the designability of the organic groups to prepare more ILs-POSS equipped with various fluorophores and enhance the fluorescent sensibility of ILs-POSS.

Fe^3+^ as an indispensable trace metal element in living organisms [22] has played an essential part in the progress of oxygen uptake and metabolism and electron transfer. If the content of Fe^3+^ exceeded the limit, it would cause disorders of the pancreas [23], the heart, the lungs, and other functional organs. On the other hand, a deficiency of Fe^3+^ could cause anemia [24]. It is known to all that many heavy metal ions such as Fe/Mn/Cu/Ni/Fe/Co/Pb/As exist in waste water that are harmful to all organisms on Earth [25]. Along with the rapid growth of mining, batteries, the paper industry, and chemical fertilizer fields, more and more heavy metal waste water is discharged directly or indirectly into the environment, causing serious influences on the ecosystem. Some researchers have demonstrated Alzheimer’s disease and Parkinson’s disease are associated with Fe^3+^. In addition, the United States Environmental Protection Agency (UEPA) and the World Health Organization have set a limit of Fe which is no more than 5.4 μmol/L [26], therefore accurate and quantitative detection of Fe^3+^ has great significance [27]. Traditional detection methods are complex and time-consuming [28]. In addition, the relevant equipment is expensive and the preparation of samples is very complicated and not recyclable. Accordingly, fluorescent sensors for the detection of Fe^3+^ have attracted much attention due to their high sensitivity, simplicity, cost-effectiveness, and fast response times [29].

Imidazolium functionalized ILs-POSS have performed strong fluorescent abilities and been employed in the detection of metal ions [6]. Nevertheless, simple compounds functionalized with one or two imidazolium groups could not possess distinct sensibility of the Fe ion, and the temperature range has also limited its application. Based on the previous research, we adopted the green chemistry approach—the thiol-ene “click” reaction to construct imidazolium-functionalized ILs based on POSS for three purposes: First, the preparation of the ILs should meet green chemistry without a metal catalyst; second, compounds containing an imidazole ring perform excellent fluorescent properties that benefit synthesizing more ILs applied to the detection field; third, we attempt to explore the differences of the ability of the ILs with various chain length in the same detection. Thereby, we constructed three novel imidazolium ILs-POSS with excellent detection abilities in Fe^3+^ in order to expand the fields of ILs and silicone materials.

## 2. Materials and Methods 

### 2.1. Materials

The starting materials (1-allylimidazole, ethyl 3-bromopropionate, ethyl 4-bromobutyrate, and ethyl 5-bromovalerate) were purchased from Aladdin Co. (Shanghai, China) and used without further purification. 2,2-dimethoxy-2-phenylacetophenone (DMPA) was purchased from the Aladdin Co. (Beijing, China) and used as received. Tetrahydrofuran (THF) and toluene were purified and distilled over sodium before use. The routine procedure was as follows: Benzophenone was used as an indicator, and sodium metal was added to the THF, which was heated until the solution was dark blue. Octa(mercaptopropyl)silsesquioxane (denoted as POSS-SH) were prepared according to a method described in the literature [30].

### 2.2. Characterization and Measurements

^1^H NMR, ^13^C NMR, and ^29^Si NMR spectra were recorded on a Bruker Avance-400 spectrometer (Bruker Co., Rheinstetten, Germany) using CDCl_3_ or a mixture of CD_3_OD and acetone-d_6_ as the solvent and without tetramethylsilane (TMS) as an internal reference. High resolution mass spectra (HRMS) were obtained in the negative mode on an Agilent Technologies 6510 Q-TOF mass spectrometer (Agilent Co., Santa Clara, CA, USA). FTIR spectra were recorded on a Bruker TENSOR-27 infrared spectrophotometer (Bruker Co., Ettlingen, Germany) via the KBr pellet technique within the wave number region from 4000 to 400 cm^−1^. Luminescence (excitation and emission) spectra of the samples were recorded with a Hitachi F-4500 fluorescence spectrophotometer (Rigaku Co., Tokyo, Japan) equipped with a monochromatic Xe lamp as an excitation source. Thermal measurements were carried out using a TA Instruments SDTQ 600 (Mettler Co., Shanghai, China). The ILs-POSS were loaded into aluminium pans, which were then heated from −150 to 40 °C, cooled to −150 °C, and finally reheated to 40°C. The heating and cooling temperature ramping rates were 10 °C/min. The differential scanning calorimetry (DSC) data reported in this paper were from the second heating cycle. TGA was performed on a Mettler Toledo TGA/DSC1 (Mettler. Co., Shanghai, China) at a heating rate of 10 °C/min from room temperature to 700 °C under N_2_ (10 mL/min) at ambient pressure. To analyze the self-assembly behaviors of the ILs-POSS by TEM observation, samples were prepared by spreading a drop of aggregated solution onto a copper grid, followed by air drying at room temperature before testing on a JEM-1011 (100 kV) electron microscope (JEOL, Tokyo, Japan).

### 2.3. General Procedures to Synthesize ILs-POSS

**Scheme 1:**
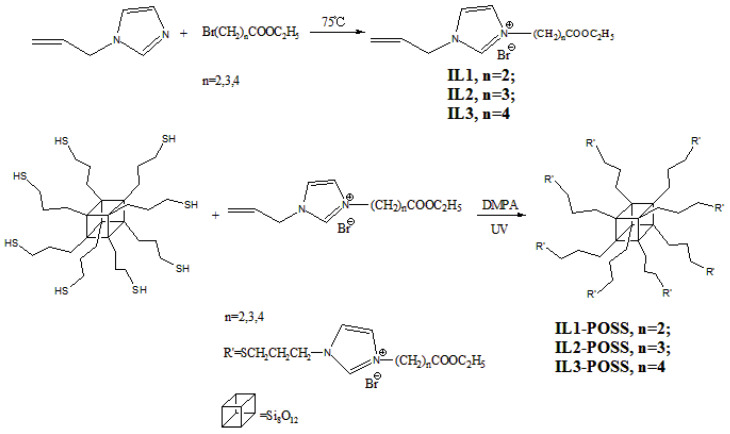
Synthesis of IL1-POSS, IL2-POSS and IL3-POSS.

The thiol-ene reaction mixture was irradiated with high-intensity UV light from a Spectroline model SB–100P/FA lamp (365 nm, 100 W, Spectroline Co., Westbury, NY, USA). As illustrated in Scheme 1, first IL1 was prepared through a quaternization reaction with 1-allylimidazole and ethyl 3-bromopropionate. Then IL1-POSS was synthesized via thiol-ene “click” reaction as follows: POSS-SH (1.06 g; 1 mmol), IL1 (2.31 g; 8 mmol), and DMPA (0.05 g; 2 wt%) were added to a transparent bottle with a 10 mL solvent mixture of CH_3_OH and CH_2_Cl_2_. The starting materials were then irradiated with a UV lamp for 15 min after dissolving completely. Finally, IL1-POSS was obtained after solvent evaporation at low pressure and vacuum drying at 60 °C for 24 h. The yield of IL1-POSS was 91%. IL2-POSS (yield: 92%) and IL3-POSS (yield: 90%) were prepared in the same way as above. 

## 3. Results

### 3.1. Synthesis and Characterization 

In this paper, we prepared a series of ILs-POSS: IL1-POSS, IL2-POSS, and IL3-POSS. The synthetic routes are described in Scheme 1.

First, IL1 (shown in the Scheme 1) was prepared via the reaction of 1-allylimidazole with ethyl 3-bromopropionate. Afterwards, IL1-POSS was synthesized via thiol-ene “click” reaction according to the route illustrated in Scheme 1. Fourier transform infrared (FTIR) spectroscopy was employed to characterize the structure and monitor the extent of the reaction. After reacting for 15 min, the disappearance of the -SH groups (γ = 2565 cm^−1^) was consistent with the appearance of peaks that were assigned to the imidazole ring at 1579 and 1446 cm^−1^. In the meantime the peaks of the carbonyl group were around 1730 cm^−1^, as shown in Figure S1. Second, the ^1^H NMR spectra of the ILs-POSS are shown in Figure S2. For IL1-POSS, whereas the characteristic -SH peak at 1.39 ppm (shown in Figure S2a) and the CH=CH_2_ peak at approximately 5.5–6.0 ppm vanished (shown in Figure S2b), a new peak appeared at 2.27 ppm and signals corresponding to the imidazole ring appeared at 7.76 and 9.97 ppm. The ^13^C NMR spectra of ILs-POSS are shown in Figure S3. Furthermore, only one peak (δ = −21.88 ppm) was detected in the ^29^Si NMR spectrum of IL1-POSS (shown in Figure S4), which demonstrated that the POSS cage remained intact during the whole reaction. Additionally, there were three peaks at m/z = 535.2002 (IL1-POSS), m/z = 535.2038 (IL2-POSS), and m/z = 535.2016 (IL3-POSS) in the mass spectra (Figure S5), which provided evidence that the targeted products were successfully synthesized.

### 3.2. Thermal Properties

**Figure 1:**
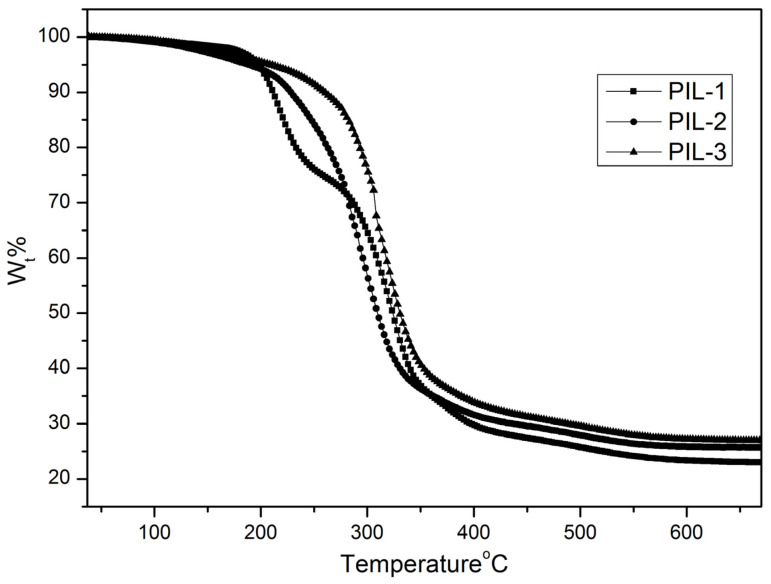
TGA curves of the ILs-POSS.

To thoroughly characterize the thermal behaviors of the three ILs-POSS, thermogravimetric analysis (TGA) and differential scanning calorimetry (DSC) were performed. The thermal decomposition temperatures (T_d_ at 5 wt%) of the ILs-POSS ranged from 200 °C to 300 °C (shown in Figure 1). For instance, in the case of IL3-POSS, the slight decrease in mass observed may be attributed to the loss of volatile compounds [16]. It is obvious that the three ILs-POSS performed excellent thermal stability, which is ascribed to two causes: one was the highly structured POSS cage formed by the Si-O-Si bond; the other one was that there existed strong ionic interactions in these ILs-POSS, conferring these molecules with wider temperature application ranges.

**Figure 2:**
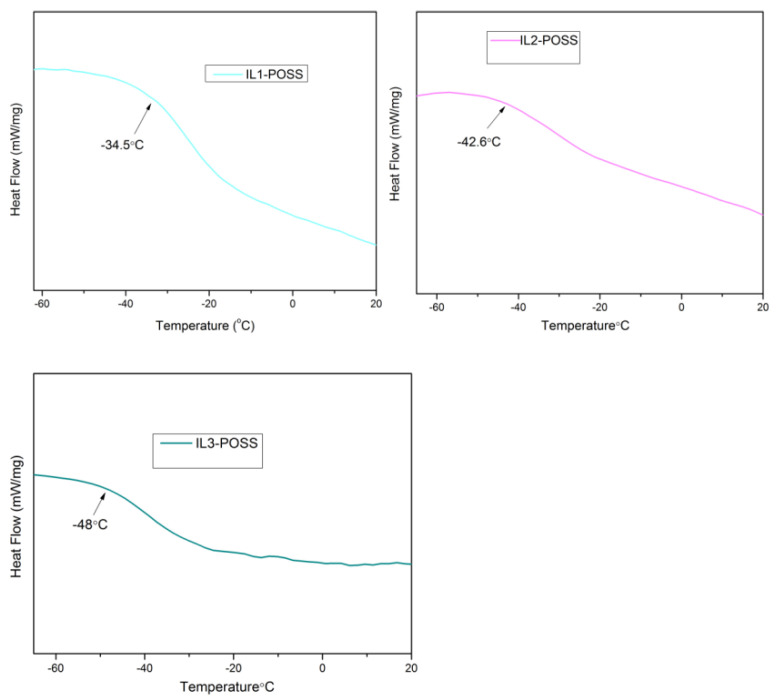
DSC curves of the ILs-POSS.

It is obvious that these three ILs-POSS all exhibited a single glass transition temperature (T_g_) (shown in Figure 2), which means there was no phase separation in the products. Additionally, IL3-POSS exhibited an endothermic peak at approximately −48°C (shown in Figure 2), whereas IL1-POSS and IL2-POSS exhibited an endothermic peak at approximately −34.5 °C and 42.6 °C, respectively. This result is in accordance with the theory that a softer polymer chains leads to a lower T_g_. Furthermore, according to the literature [29], the category and size of the anion also has a certain effect on the variation of T_g_ when there is a larger difference between the size of cations and anions [18]; the T_g_ is lower and apparently the data demonstrated the theory. Therefore, it is realistic to introduce POSS into the structure of ILs-POSS for the improvement of the temperature application ranges. 

### 3.3. Self- Assembly Behaviors

**Figure 3:**
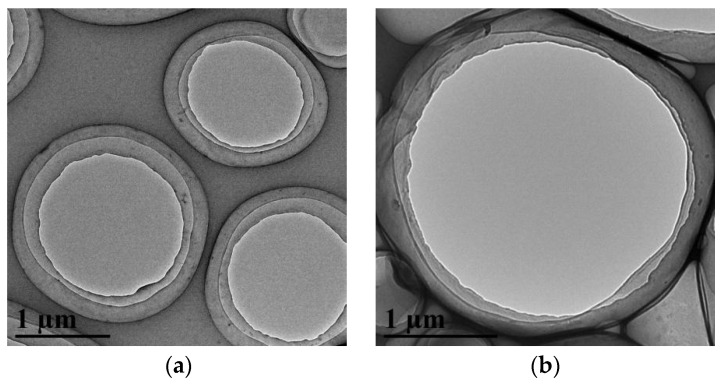
(**a**) IL3-POSS dissolved in mixed solvent (acetone:H_2_O = 1:9) and (**b**) IL3-POSS dissolved in mixed solvent (acetone:H_2_O = 1:1).

Self-assembly behaviors have great significance on replicating the complex process of the evolution of life [5]. Taken IL3-POSS as an example: Our study showed that different aggregation behaviors occurred when the IL3-POSS was in different solvents (shown in Figure 3). When IL3-POSS was in the good solvent, it is obvious that a dendritic IL centered on the POSS cage was generated by the combination of the imidazolium with POSS-SH. These dendritic ILs-POSS were in free arrangements when they were in favorable solvents [31], whereas in some certain solvents, self-assembly behavior occurred [32], and spherical vesicles were formed with anions on the outside and a POSS cage [30] on the inside. In Figure 3a,b, IL3-POSS formed into 2 μm spherical vesicles in mixed solvents. Furthermore, aggregation of the cations [33] and anions was generated by the strong electrostatic interactions existing in the ILs-POSS. These data suggest that the spherical vesicles represent the lowest energy state reached through electrostatic interactions. It is known to all that one the most important applications of vesicles is to simulate biofilms. Based on the above data, our study provides a new approach of the bionic research through the study of the IL3-POSS reported.

### 3.4. Optical Properties

**Figure 4:**
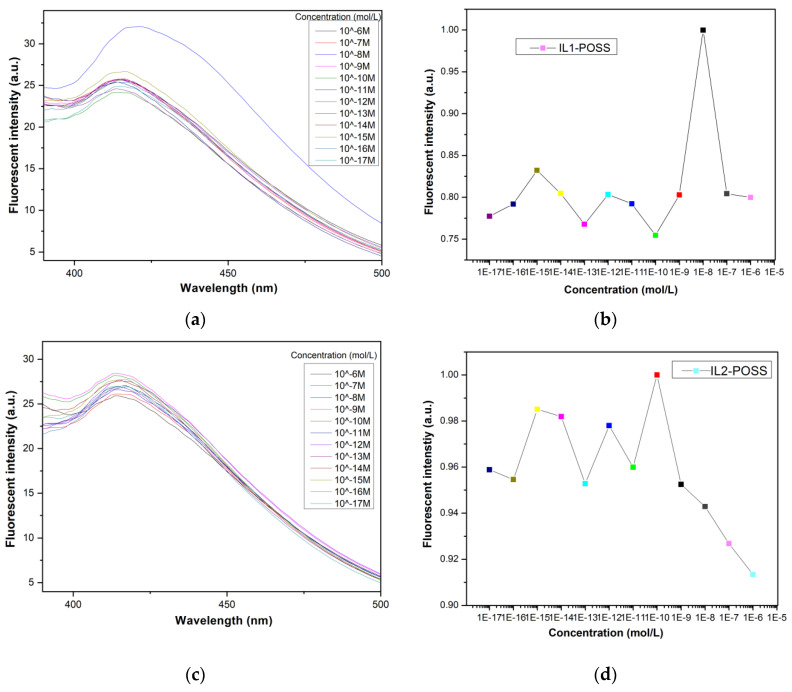

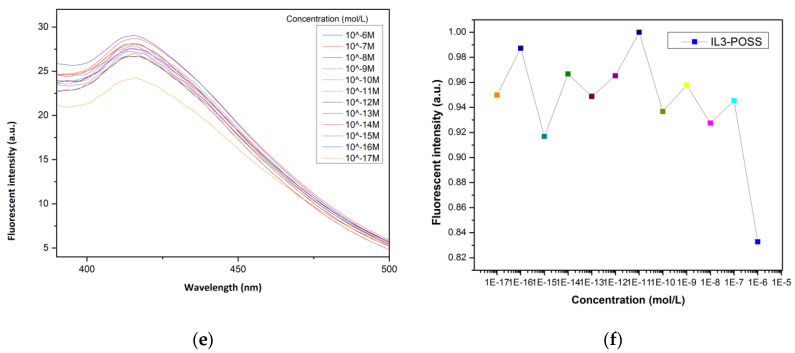
(**a**–**e**) are emission spectra of IL1-POSS, IL2-POSS and IL3-POSS, respectively at different concentrations. (**b**–**f**) are plots of normalized fluorescent intensity versus concentrations of the IL1-POSS, IL2-POSS and IL3-POSS, respectively. λ_ex_ = 365nm.

For further characterization, we studied the fluorescent properties of the ILs-POSS. When excited at 365 nm, the three ILs-POSS exhibited emission bands centered around 410 nm, as shown in Figure 4a,c,e. It is attributed to two causes: Firstly, an imidazole ring is a five-membered heterocyclic compound containing two nitrogen atoms, which forms a big π bond. In addition, there is a lone pair of electrons in the *sp*^2^ orbital of an N atom. When an imidazole ring linked with various electron-donating or electron-withdrawing groups, intramolecular charge transfer occurred. In the meantime, silicon atoms possessed five empty 3*d* orbitals, which were conductive to the coordination between silicon atoms and lone pair electrons. It led to the splitting of the 3*d* orbitals of silicon atoms and the rearrangement of the electrons in the splitting orbitals. Secondly, the Si→S coordination bond facilitated the splitting of the 3*d* orbital of Si atoms and the rearrangement of the electrons that led to a *d*-*d* transition. Accordingly, the above reasons had great influence on the fluorescent properties of the ILs-POSS. 

Take IL1-POSS, for example. In Figure 4a,b, when excited at 365 nm, the fluorescent intensity was very weak around the concentration of 10^−17^ mol/L. As the concentration increased to 10^−8^ mol/L, the fluorescent intensity was 1.3 times the initial intensity. On the other hand, as the concentration increased, the fluorescent intensity declined, and the intensity at a concentration of 10^−6^ mol/L was approximate to that of the concentration at 10^−17^ mol/L. IL2-POSS and IL3-POSS exhibited similar properties with IL1-POSS, that is, the fluorescent intensity first enhanced then declined with the increasing concentration.

## 4. Discussion

### 4.1. ILs-POSS in the Detection of Fe^3+^

**Figure 5:**
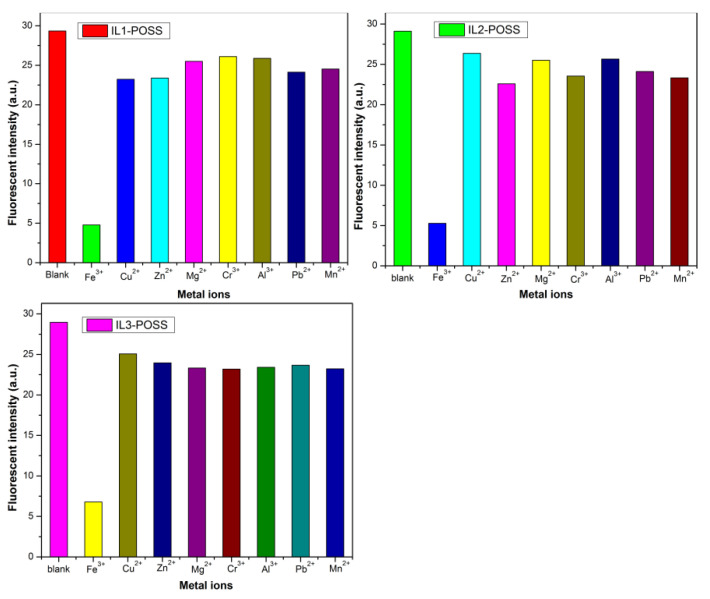
Fluorescent intensity of ILs-POSS (10^−5^ mol/L) with different metal ions (10^−2^ mol/L).

It is known to all that metal ions have great influences on organism and environmental systems, therefore it is essential to achieve efficient metal ion detection. Based on the above data, we explored the potential application of ILs-POSS fluorescent sensors in detecting metal ions, including Fe^3+^, Cu^2+^, Zn^2+^, Mg^2+^, Cr^3+^, Al^3+^, Pb^2+^, and Mn^2+^. As shown in Figure 5, it is obvious that ILs-POSS exhibited effective detection ability in Fe^3+^ relative to other metal ions. The sensing studies were performed by recording the changes with excitation at 365 nm. 

**Figure 6:**
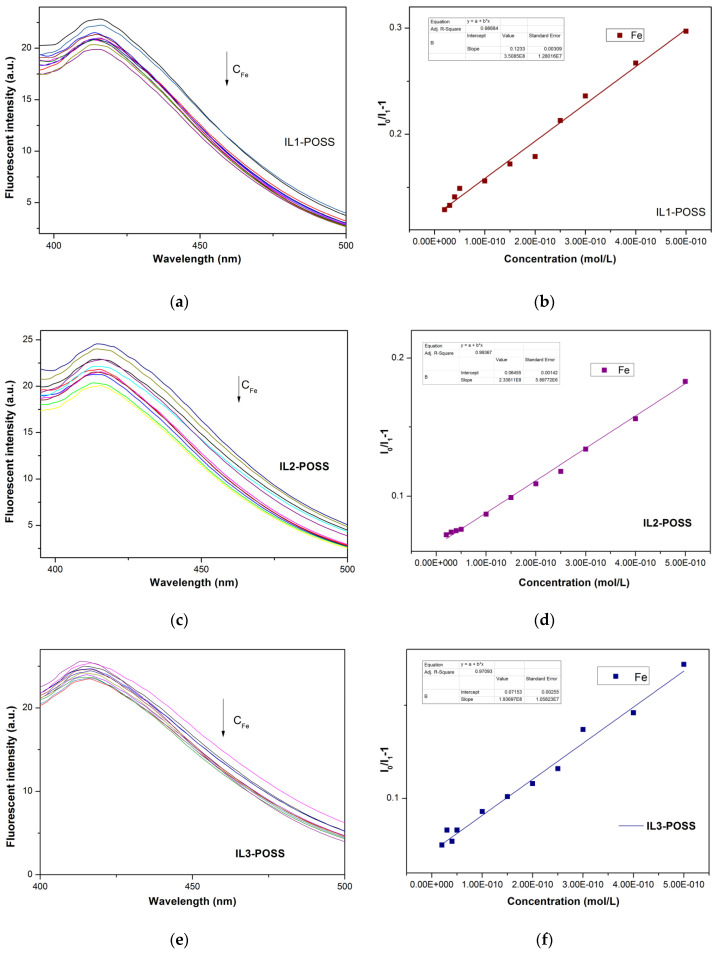
(**a**,**c**,**e**) are fluorescent quenching spectra of ILs-POSS (10^−7^ mol/L) with Fe^3+^ (10^−7^ mol/L) in different concentrations (the dosage of Fe^3+^ was 2 μL, 3 μL, 4 μL, 5 μL, 10 μL, 15 μL, 20 μL, 25 μL, 30 μL, 40 μL, and 50 μL), and (**b**,**d**,**f**) are Stern-Volmer plots of ILs-POSS for detecting Fe^3^^+^.

In order to further characterize the detection ability in Fe^3+^ of the three ILs-POSS, we studied the fluorescent intensity of ILs-POSS with different concentration of Fe^3+^, and the Stern-Volmer plots were also obtained, as shown in Figure 6.

It is apparent that the fluorescent intensity declined along with the concentration of Fe^3+^ increasing. The fluorescent quenching mechanism of metal ions with fluorescent materials were ascribed to two reasons: one was the traditional electron transfer mechanism of the electron transfer from the imidazole rings of ILs-POSS to Fe^3+^ occurred among the molecules; the other one was the electron transition between imidazole rings and 3D vacant orbitals of Si atoms. Accordingly, the coexistence of the electron transfer effect and the electron transition between the imidazole rings and Si atoms led to this fluorescent quenching phenomenon. 

There are two important parameters for the detection of metal ions: *K_sv_* and LODs, which are obtained by the following equations [34]:

I_0_/I = 1 + *K_sv_*[c] (1)

LODs = 3 × σ/*K* (2)

where σ: standard deviation of blank sample and c: the concentration of Fe^3+^.

Illustrated by the Stern–Volmer curves shown in Figure 5, we obtained the data that *K_sv_*1 = 3.51 × 10^8^ M^−1^, *K_sv_*2 = 2.34 × 10^8^ M^−1^, and *K_sv_*3 = 1.94 × 10^8^ M^−1^, and according to the formula LOD = 3 × σ/*K*_sv_ (σ1 = 9.25, σ2 = 9.38, σ3 = 9.51), we obtained the LOD value of the three ILs-POSS, which were LOD_1_ = 7.91 × 10^−8^ M, LOD_2_ = 1.2 × 10^−7^ M, and LOD_3_ = 1.47 × 10^−7^ M. In conclusion, the three ILs-POSS could be used to detect Fe^3+^ at extremely low concentrations.

Based on the data above, the ILs-POSS performed excellent fluorescent ability in detecting Fe^3+^, which demonstrates that ILs-POSS have potential application in the field of fluorescent sensors.

### 4.2. Efficient Fluorescent Paper for Detecting Fe^3+^

**Figure 7:**
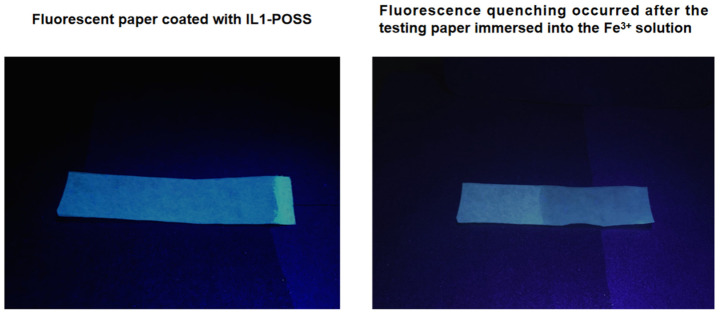
Under a UV lamp (365 nm), photographs of fluorescent paper immobilized with IL1-POSS before and after being immersed into the Fe^3+^ aqueous solution.

For the sake of intuitive observation of ILs-POSS in detecting Fe^3+^, the ILs-POSS were immobilized on the testing paper to obtain the fluorescent paper shown in Figure 7.

It is noteworthy that fluorescence was quenched after the testing papers were immersed in the Fe^3+^ aqueous solution. This phenomenon suggested that the three ILs-POSS reported were equipped with the potential ability to be employed as the fluorescent sensors in the field of detecting metal ions. These three ILs-POSS were distinct from other traditional ionic liquids. Firstly, it was more in accordance with the concept of “green chemistry” to adopt the approach of the thiol-ene “click” reaction, which does not consist of metal catalysts; secondly, the introduction of an Si atom into the product structures was beneficial to the enhancement of fluorescent intensity; thirdly, with POSS as the core of the structure, the application temperature range of the ILs-POSS was expanded.

## 5. Conclusions

All in all, the three ILs-POSS were synthesized in high yields through thiol-ene “click” reaction and characterized by comprehensive measurements. The ILs-POSS reported in this paper exhibited excellent thermal stabilities and low glass transition temperatures profiting from the highly structured POSS cage. Furthermore, the ILs-POSS presented spherical vesicle structures in selected solvents due to the unique amphiphilic nature and the strong electrostatic interactions among the molecules. Additionally, the fluorescent intensity of the ILs-POSS also performed excellently, not only owing to the imidazole ring, but also to the Si→S coordination bond. Based on the above data, we found the application in detecting Fe^3+^ of the three ILs-POSS, and the results demonstrated that the ILs-POSS performed excellent fluorescent sensibilities in detecting Fe^3+^ and have great potential to act as the fluorescent sensors in the field of detecting metal ions. In conclusion, this report described the synthesis of the novel ILs-POSS through thiol-ene “click” reaction and provided a potential application as fluorescent sensors. It is expected that more and more functionalized ILs-POSS can be produced via green chemistry reaction and applied as practical sensors in the future.

## Data Availability

Data is contained within the article or supplementary material. The data presented in this study are available in article.

## Supplementary Materials

The following are available online at https://www.mdpi.com/2073-4360/13/2/196/s1, Figure S1: FTIR spectra of ILs-POSS, Figure S2: ^1^H spectra of POSS-SH and ILs-POSS, Figure S3: ^13^C spectra of IL3-POSS, Figure S4: ^29^Si spectrum of IL1-POSS, S5: HRMS spectra of IL1-POSS, IL2-POSS and IL3-POSS.
