# Supplementary material for: New Functionalized Ionic Liquids Based on POSS for the Detection of Fe3+ Ion
Source: Polymers (Basel). 2021 Jan 7;13(2):196. doi: 10.3390/polym13020196 (PMC7827438; doi:10.3390/polym13020196)
Supplement: Supplementary file 1 [file polymers-13-00196-s001.pdf]

polymers

New Functionalized Ionic liquids Based on POSS for the Detection of Fe^{3+} Ion

Wensi Li[1], Shengyu Feng[1,2*]

[1] Key Laboratory of Special Functional Aggregated Materials Ministry of Education, School of Chemistry and Chemical Engineering, Shandong University, Jinan 250100, China; anterlina@163.com (W.L.)
[2] National Engineering Technology Research Centre for Colloidal Materials, Shandong University, Jinan 250100, China
* Correspondence: fsy@sdu.edu.cn (S.F.); Tel.: +86-0531-88364866 (S.F.)

Experimental Section

Materials.

The starting materials (1-allylimidazole, ethyl 3-bromopropionate, ethyl 4-bromobutyrate, ethyl 5-bromovalerate) were purchased from Aladdin Co., Shanghai, China and used without further purification. 2,2-dimethoxy-2-phenylacetophenone (DMPA) was purchased from the Aladdin Co. (Beijing, China) and used as received. Tetrahydrofuran (THF) and toluene were purified according to a routine procedure and distilled over sodium before use. Octa(mercaptopropyl)silsesquioxane (denoted as POSS-SH) were prepared according to a method described in the literature[1].

Characterization and measurements

The thiol-ene reaction mixture was irradiated with high-intensity UV light from a Spectroline model SB-100P/FA lamp (365 nm, 100 W, Spectroline Co., Westbury, NY, USA). ^{1}H NMR, ^{13}C NMR, and ^{29}Si NMR spectra were recorded on a Bruker Avance-400 spectrometer using $CDCl_3$ or a mixture of CD_3OD and acetone-d_6 as the solvent and without tetramethylsilane (TMS) as an internal reference. High resolution mass spectra (HRMS) spectra were obtained in the negative mode on an Agilent Technologies 6510 Q-TOF mass spectrometer (Agilent Co., Santa Clara, CA, USA). FT-IR spectra were recorded on a Bruker TENSOR-27 infrared spectrophotometer (Bruker Co., Ettlingen, Germany) via the KBr pellet technique within the wave number region from 4000 to 400 cm-1. Luminescence (excitation and emission) spectra of the samples were recorded with a Hitachi F-4500 fluorescence spectrophotometer (Rigaku Co., Tokyo, Japan) equipped with a monochromatic Xe lamp as an excitation source. Thermal measurements were carried out using a TA Instruments SDTQ 600. The ILs-POSS were loaded into aluminium pans, which were then heated from -150 to 40°C, cooled to -150°C, and finally reheated to 40°C. The heating and cooling temperature ramping rates were 10°C/min. The DSC data are reported in this paper from the second heating cycle. TGA was performed on a Mettler Toledo TGA/DSC1 at a heating rate of 10°C/min from room temperature to 700°C under N_2 (10 mL/min) at ambient pressure.

General procedures to synthesize ILs-POSS

IL1 was prepared through a quaternisation reaction. IL1-POSS was synthesized via a classic procedure as illustrated in Scheme 1. POSS-SH (1.06 g; 1 mmol), IL1 (2.31 g; 8 mmol), and DMPA (0.05 g; 2 wt%) were added to a transparent bottle with a 10 mL solvent mixture of CH_3OH and CH_2Cl_2. The starting materials were then irradiated with a UV lamp for 15 min after dissolving completely. Finally, IL1-POSS was obtained after solvent evaporation at low pressure and vacuum drying at 60°C for 24 h. IL2-POSS and IL3-POSS are prepared in the same way above.

Data of IL1-POSS:
^{1}H NMR (500 MHz, CD_3OD) δ 9.97, 8.01, 7.99, 7.98, 4.43, 3.97, 3.96, 2.99, 2.59, 2.58, 2.27, 2.26, 1.96, 1.88, 1.55, 1.54, 1.16, 1.15, 0.92.
^{13}C NMR (126 MHz, MeOD) δ 170.55, 136.07, 122.69, 60.89, 48.12, 47.94, 47.78, 47.44, 47.26, 33.97, 27.44, 19.16, 12.62 , 10.62.
^{29}Si NMR (79 MHz, MeOD) δ -21.88.
HRMS: 535.2002
Data of IL2-POSS:

^{1}H NMR (500 MHz, CD_3OD) δ 9.99, 8.03, 8.01, 7.90, 7.74, 7.29, 6.97, 6.95, 4.40, 4.39, 4.38, 4.37, 4.34, 4.33, 4.32, 4.30, 4.27, 4.25, 4.24, 4.18, 4.13, 4.12, 4.10, 4.09, 3.89, 2.61, 2.60, 2.59, 2.53, 2.52, 2.50, 2.45, 2.44, 2.43, 2.40, 2.38, 2.37, 2.31, 2.26, 2.20, 2.18, 2.14, 2.13, 2.09, 2.06, 1.98, 1.92, 1.91, 1.70, 1.69, 1.57, 0.98, 0.97, 0.77.
^{13}C NMR (126 MHz, CD_3OD) δ 172.50, 136.66, 129.65, 122.52, 60.47, 48.99, 48.73, 48.12, 48.06, 47.43, 47.09, 33.94, 27.67, 13.18, 10.61.
HRMS: 535.2038
Data of IL3-POSS:
^{1}H NMR (500 MHz, CD_3OD) δ 9.98, 9.38, 8.04, 7.99, 7.71, 7.31, 4.27, 4.24, 4.20, 4.18, 4.17, 4.12, 4.08, 2.59, 2.53, 2.52, 2.49, 2.41, 2.39, 2.38, 2.35, 2.34, 2.32, 2.32, 2.27, 2.26, 2.09, 2.08, 1.62, 1.58, 1.56, 1.55, 1.53, 1.24, 1.18, 1.10, 0.97, 0.78.
^{13}C NMR (126 MHz, CD_3OD) δ 174.89, 138.20, 135.42, 134.63, 131.20, 129.90, 128.09, 124.03, 104.96, 61.76, 50.74, 35.47, 34.26, 30.57, 29.19, 14.76.
HRMS:535.2016

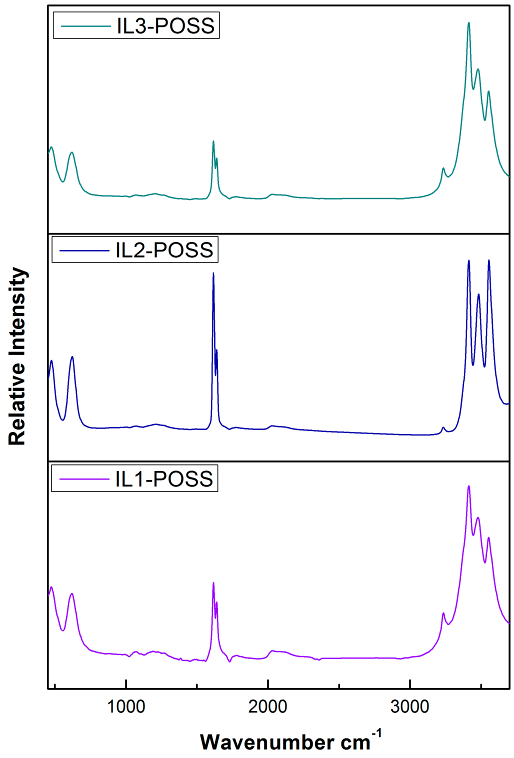

Figure S1. FTIR spectra of ILs-POSS

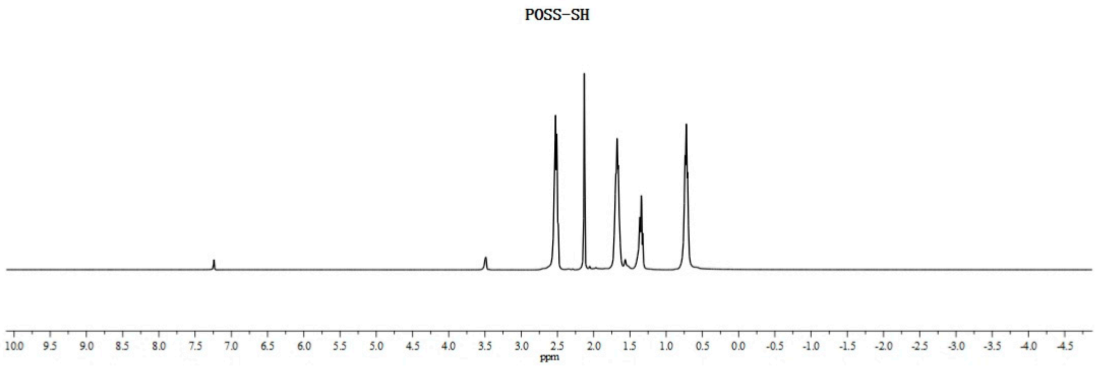
POSS-SH
10.0
9.5
9.0
8.5
8.0
7.5
7.0
6.5
6.0
5.5
5.0
4.5
4.0
3.5
3.0
2.5
2.0
1.5
1.0
0.5
0.0
-0.5
-1.0
-1.5
-2.0
-2.5
-3.0
-3.5
-4.0
-4.5
ppm

(a)

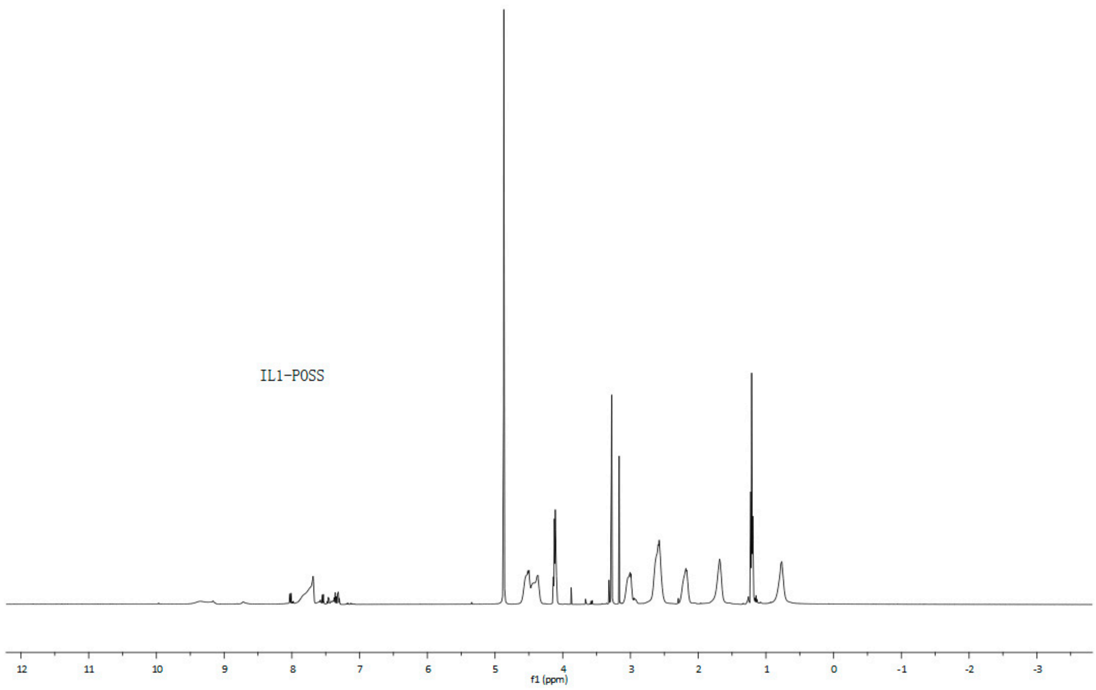
IL1-POSS
12
11
10
9
8
7
6
5
4
3
2
1
0
-1
-2
-3
f1 (ppm)

(b)

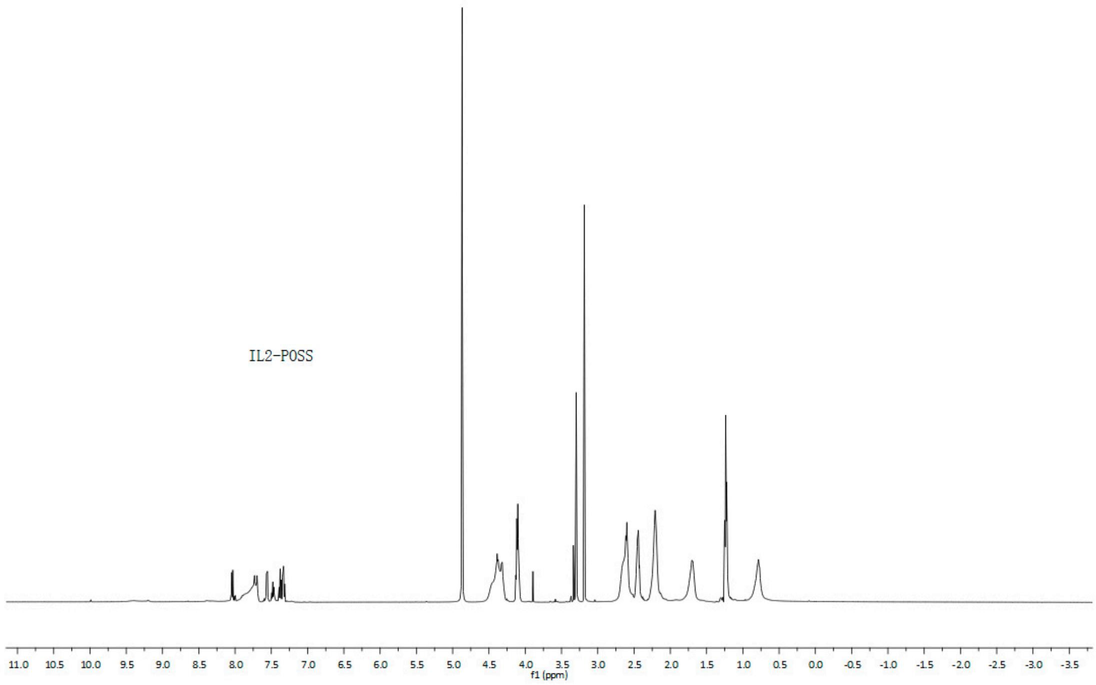

(c)

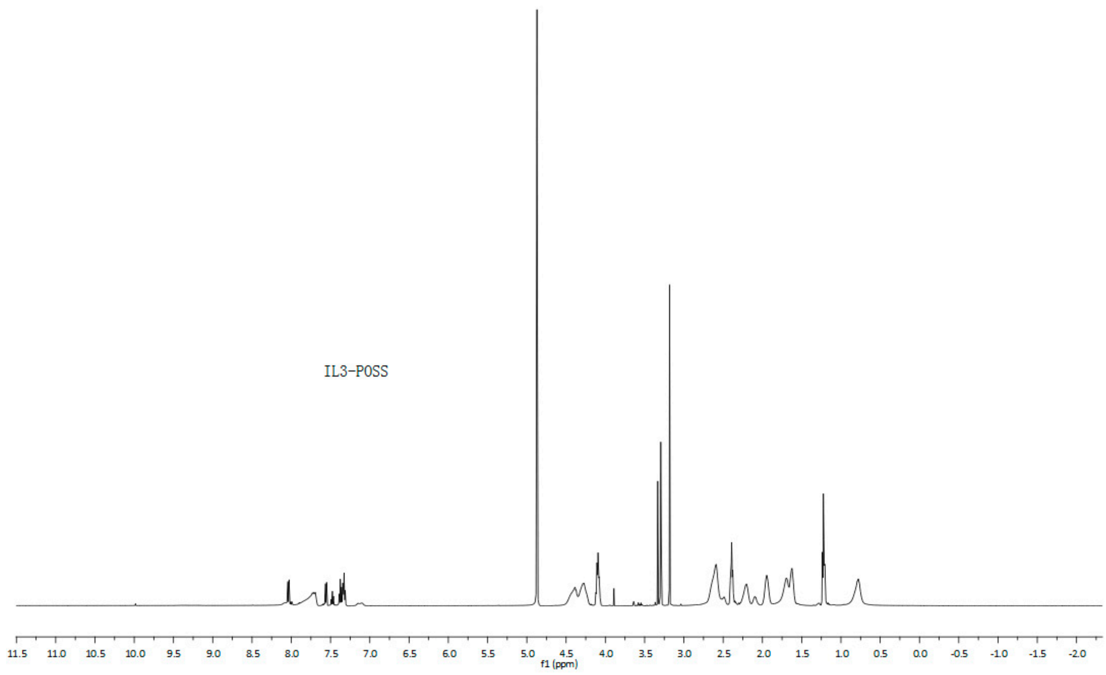

(d)

Figure 2. ^{1}H **spectra of POSS-SH and ILs-POSS.**

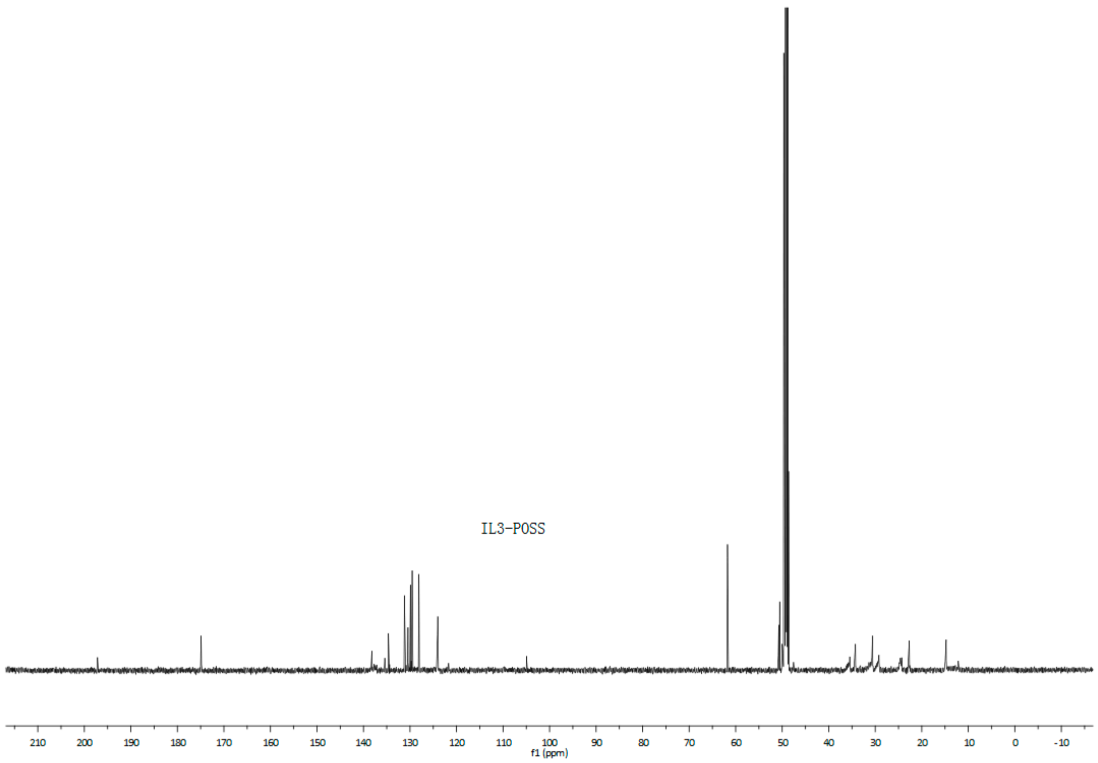

Figure 3. ^{13}C spectra of IL3-POSS

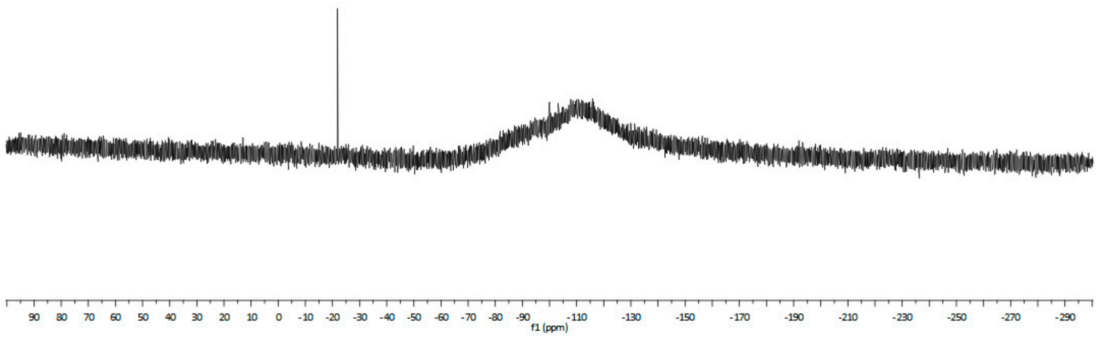

Figure 4. ^{29}Si spectrum of IL1-POSS

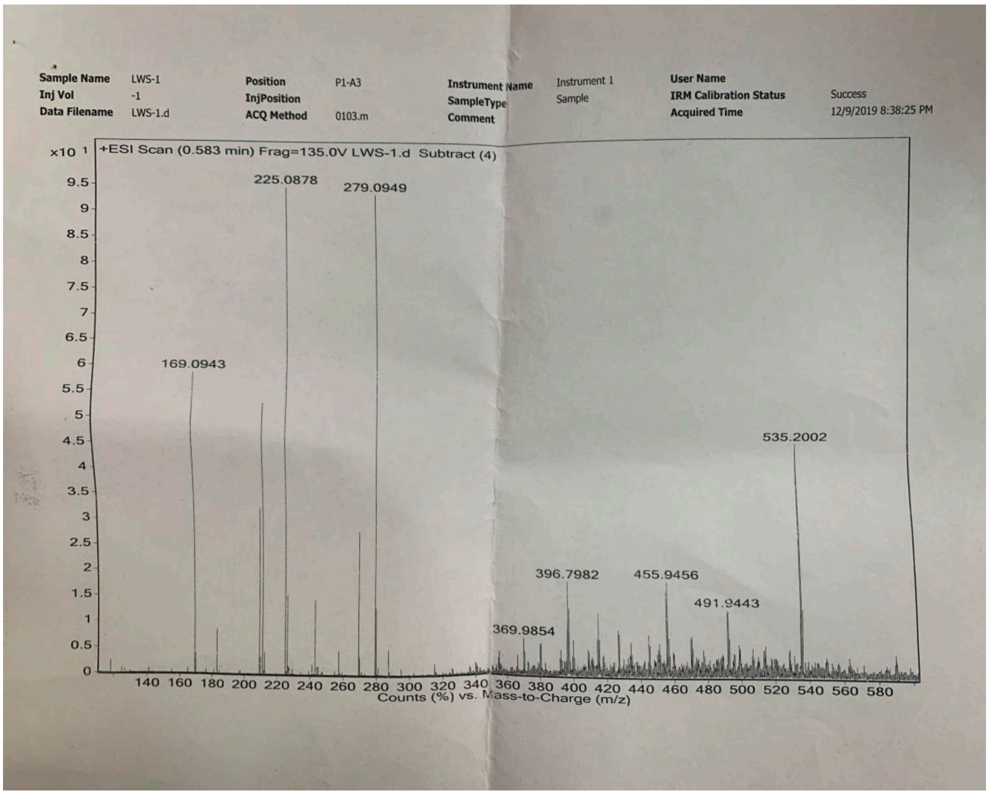

Sample Name LWS-1
Inj Vol -1
Data Filename LWS-1.d
Position P1-A3
InjPosition
ACQ Method 0103.m
Instrument Name Instrument 1
SampleType Sample
Comment
User Name
IRM Calibration Status Success
Acquired Time 12/9/2019 8:38:25 PM
x10 1
+ESI Scan (0.583 min) Frag=135.0V LWS-1.d Subtract (4)
225.0878
279.0949
169.0943
535.2002
396.7982
455.9456
491.9443
369.9854
Counts (%) vs. Mass-to-Charge (m/z)

(a)

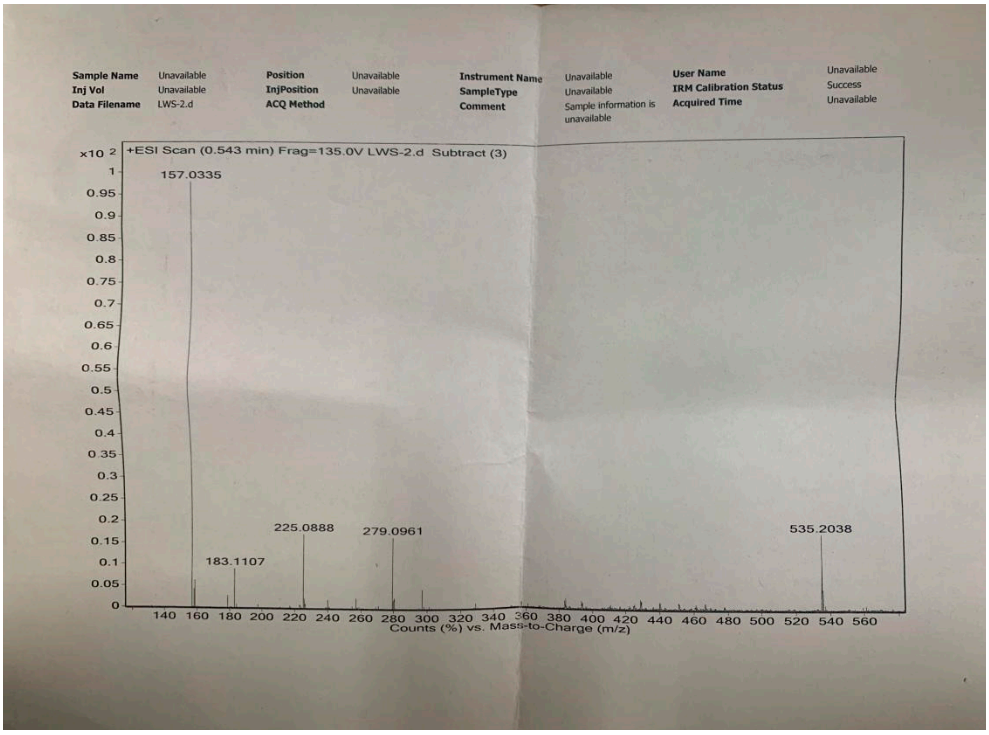

Sample Name Unavailable
Inj Vol Unavailable
Data Filename LWS-2.d
Position Unavailable
InjPosition Unavailable
ACQ Method
Instrument Name Unavailable
SampleType Unavailable
Comment Sample information is unavailable
User Name Unavailable
IRM Calibration Status Success
Acquired Time Unavailable
x10 2
+ESI Scan (0.543 min) Frag=135.0V LWS-2.d Subtract (3)
157.0335
225.0888
279.0961
183.1107
535.2038
Counts (%) vs. Mass-to-Charge (m/z)

(b)

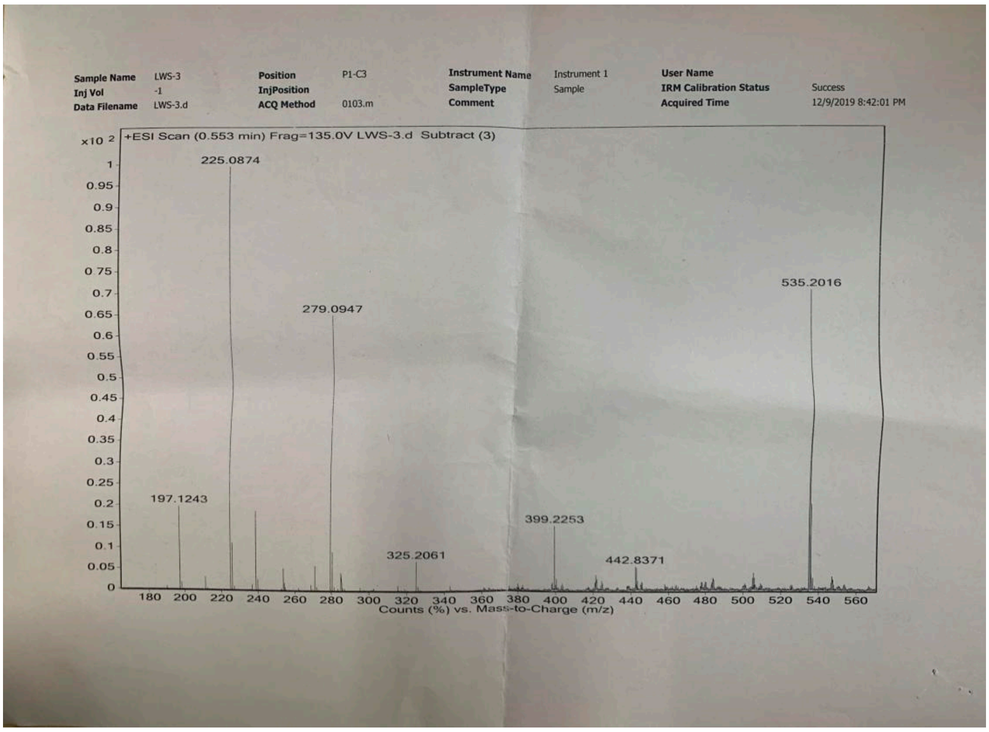

(c)

Figure S5. HRMS spectra of IL1-POSS, IL2-POSS and IL3-POSS.

References

1. Li, L.; Liu, H. Rapid Preparation of Silsesquioxane-Based Ionic Liquids. *Chemistry* **2016**, *22*, 4713-4716, doi:10.1002/chem.201600186.
